# Editorial: Exercise as a Countermeasure to Human Aging, Volume II

**DOI:** 10.3389/fphys.2022.878983

**Published:** 2022-03-21

**Authors:** Lawrence D. Hayes, Martin Burtscher, Bradley T. Elliott

**Affiliations:** ^1^ Institute of Clinical Exercise and Health Science, School of Health and Life Sciences, University of the West of Scotland, Glasgow, United Kingdom; ^2^ Department of Sport Science, Medical Section, University of Innsbruck, Innsbruck, Austria; ^3^ Translational Physiology Research Group, University of Westminster, London, United Kingdom

**Keywords:** ageing, exercise, sarcopenia, HIIT (high intensity interval training), editorial

Tis impossible to be sure of anything but death and taxes (Christopher Bullock in the Cobbler of Preston, 1716). We are inclined to agree with Bullock concerning the certainly of death and in this context, aging is ubiquitous amongst humans which results in a deterioration of physiological function and an inevitable march towards death. However, physical activity and exercise are known to exert positive effects on health and wider physiological function *via* multiple complex and interacting mechanisms (that have not yet been completely defined).

In volume II of this Research Topic, 10 articles covered the interplay between exercise and aging, utilizing approaches that spanned molecular, physiological, and population scale approaches, in both healthy older populations and certain disease subsets. This work builds on our previous work *Exercise as a Countermeasure to Human Aging, Volume I* and it is a pleasure to note continued progress in this field, and the range of methodological approaches authors have used.

## High Intensity Interval Training

A classical exercise physiology approach of a short-term training programme over weeks-to-months in humans was performed by Hayes et al. These authors provided preliminary evidence that in a group of previously sedentary sexagenarians, moderate-intensity aerobic exercise and HIIT lowered interleukin-6 (IL-6) [and possible high sensitivity C-reactive protein (hsCRP)]. Another observation was that age-matched masters athletes had lower markers of inflammaging than the sedentary cohort through the investigation, emphasizing lifetime exercise habits are anti-inflammatory, but it is never too late to start and even after 30 years of sedentariness, inflammation was reduced after only 6 weeks of exercise. The same research group subsequently conducted a scoping review into the effects of HIIT on phenotypical characteristics of sarcopenia (Hayes et al.), with muscle function, muscle quantity, and physical performance generally improved, although there was scant literature in the oldest old (≥80 years of age), or those already sarcopenic. Therefore, more studies are needed in this population.

## Performance in an Aging Demographic

From a population health viewpoint, increased lifelong activity, not just short-term exercise interventions, are needed. Thus, there has been much recent interest in examining highly trained masters athletes as a physiological model of successful aging (Pollock et al., 2015; Elliott et al., 2017; Hayes et al., 2020). This Research Topic included six reports on lifelong exercising cohorts, and five examined performance of older adults in competition, with all (unfortunately!) observing poorer performance with advanced age. Firstly, Ganse et al. examined rate of performance decline in those over the age of 100 years of age between 2006 and 2019. In summary, the rate of decline was 2.53 times steeper in centenarians as is 80–96-year-olds from Sweden (previously published data from the same group). The 100 m sprint performance displayed the greatest difference between centenarians and non-centenarians, whereby centenarian performance decreased eight times steeper than in non-centenarians. The smallest differences in performance declines were in the discus and the javelin throw (1.54 and 1.27 times steeper respectively), suggesting sprint performance is most susceptible to deterioration by advanced age. Secondly, in an article from the same group Moser et al. examined pacing variation for the 200 and 400 m individual medley in age groups up to ≥75 years of age. As with the Ganse et al. article previously described, Moser and colleagues observed an increase in race time (i.e., a worsening of performance) as participant age increased. Pacing variance, using the coefficient of variance (CV) of each split exhibited greater variation in males and females in the older age groups. Pacing variation was greater in males than females, suggesting females swim a more consistent pace during competition. That said, both female and male master swimmers showed a final end spurt (i.e., the final split was the fastest) in both 200 and 400 m individual medley. Thirdly, the same research group examined historical data of the Berlin Marathon to examine whether environmental condition influenced performance in age group runners (Knechtle et al.). In a group of 869,474 valid finisher records, older age groups ran slower. Although higher daily maximum temperatures were associated with better performance in the best (and youngest) runners, increased daily maximum temperatures decreased race performance in age group marathoners from age group 35–40 years and older with no differences between the sexes. In the fourth and final paper from this research group, Reusser et al. conducted another historical analysis of the Berlin Marathon and again observed older age groups ran slower. However, an important finding was that the number of finishers increased for both women and men runners over the decades, from 236 to 8 men and women respectively in 1974, to 28,373 and 12,268 men and women respectively in 2018. This effect was manifest across all age groups, except for male athletes aged 20–49 years and athletes of both sexes above 79 years old. All these results point to an environment that is supportive of exercise participation in older age, but performance declines are inevitable. One further aspect of interest would be to examine the influence of training programme variables (i.e., volume, intensity, frequency) on the rate of performance declines. However, this would require big data approaches and continual training recording, which has only recently become possible (since cloud storage became commonplace).

## From Big Data to Microvesicles, Microarchitecture, and microRNA

Kyriakidou et al. observed that extracellular vesicle response to muscle damaging exercise (high intensity eccentric exercise) was similar in young and old participants, suggesting a preserved means for inter-tissue crosstalk in older age following muscle damage. Using the ovariectomized mouse model of postmenopausal bone loss, Zhao et al. compared downhill running, and downhill running plus an anti-irisin receptor agent, compared to controls for bone mineral density (BMD). These authors demonstrated that downhill running improved cortical and trabecular volumetric BMD and microarchitecture compared to non-exercised ovariectomized mice. However, injection with an anti-irisin receptor agent weakened the exercise effect. These authors concluded that exercise is a potent osteogenic factor and blocking of irisin receptor signalling ameliorated this effect. Thus, irisin plays a role in regulating bone response to exercise through Akt/b-catenin pathways. Considering microRNAs (mRNAs), Garai et al. made the important observation that there was considerable overlap between exosomal mRNAs (exomiRs) of young participants on a 6 month exercise intervention, and lifelong (>25 years) exercisers. Using Kyoto Encyclopedia of Genes and Genomes pathway analysis, a large portion of these exomiRs target chronic diseases such as cancer, neurodegenerative and metabolic diseases, and viral infections. As such, exomiRs may be one of the mechanisms by which exercise prevents several chronic diseases, the emerging possibility of exomiRs as a therapeutic target is exciting.

Finally, science is, and always should be, self-correcting. We applaud Garai et al. for their corrigendum correcting an mis-acknowledgement of funding source. An error of this type would unlikely be noticed, but it was important to acknowledge correctly.

As we move towards a goal of personalised treatments and medicines, it is interesting to speculate what might fill the pages of a future *Exercise as a Countermeasure to Human Ageing, Vol III*. In the editorial accompanying our first volume, we noted the representation of sexes between published work, and in this volume it is pleasing to see that half of the human studies report both males and females and sub-group results by sex, a step towards best practice recommendations (Elliott-Sale et al., 2021). As we move towards a truly personalised future, studies that account for sex, ethnic, lifelong environmental (and indeed transgenerational (Fitz-James and Cavalli, 2022) differences and their influences on personalising exercise guidelines are called for

These results show the uses of exercise physiology to continue to probe the basic biology of aging, and further suggest emerging findings that inter-tissue cross-talk during and after physical activity is preserved with age. Exercise may truly be a countermeasure to biological human aging.
